# Foliar-Applied Potassium Silicate Coupled with Plant Growth-Promoting Rhizobacteria Improves Growth, Physiology, Nutrient Uptake and Productivity of Faba Bean (*Vicia faba* L.) Irrigated with Saline Water in Salt-Affected Soil

**DOI:** 10.3390/plants10050894

**Published:** 2021-04-28

**Authors:** Emad M. Hafez, Hany S. Osman, Usama A. Abd El-Razek, Mohssen Elbagory, Alaa El-Dein Omara, Mohamed A. Eid, Salah M. Gowayed

**Affiliations:** 1 Department of Agronomy, Faculty of Agriculture, Kafrelsheikh University, Kafr El-Sheikh 33516, Egypt; 2 Department of Agricultural Botany, Faculty of Agriculture, Ain Shams University, Hadayek Shobra, P.O. Box 68, Cairo 11241, Egypt; hany_osman1@agr.asu.edu.eg; 3 Agronomy Department, Faculty of Agriculture, Tanta University, Tanta 31527, Egypt; usama.eldesouky@agr.tanta.edu.eg; 4 Department of Biology, Faculty of Science and Arts, King Khalid University, Mohail Assir 61321, Saudi Arabia; mhmohammad@kku.edu.sa; 5 Department of Microbiology, Soils, Water and Environment Research Institute, Agricultural Research Center, Giza 12112, Egypt; alaa.omara@yahoo.com; 6 Agronomy Department, Faculty of Agriculture, Fayoum University, Fayoum 63514, Egypt; mam11@fayoum.edu.eg; 7 Department of Botany, Faculty of Agriculture, Suez Canal University, Ismailia 41522, Egypt; salahgowed@yahoo.com; 8 Department of Biology, College of Sciences, University of Jeddah, Jeddah 21589, Saudi Arabia

**Keywords:** saline water, PGPR, potassium silicate, soil enzymes, ESP, seed yield, nutrient uptake

## Abstract

The continuity of traditional planting systems in the last few decades has encountered its most significant challenge in the harsh changes in the global climate, leading to frustration in the plant growth and productivity, especially in the arid and semi-arid regions cultivated with moderate or sensitive crops to abiotic stresses. Faba bean, like most legume crops, is considered a moderately sensitive crop to saline soil and/or saline water. In this connection, a field experiment was conducted during the successive winter seasons 2018/2019 and 2019/2020 in a salt-affected soil to explore the combined effects of plant growth-promoting rhizobacteria (PGPR) and potassium (K) silicate on maintaining the soil quality, performance, and productivity of faba bean plants irrigated with either fresh water or saline water. Our findings indicated that the coupled use of PGPR and K silicate under the saline water irrigation treatment had the capability to reduce the levels of exchangeable sodium percentage (ESP) in the soil and to promote the activity of some soil enzymes (urease and dehydrogenase), which recorded nearly non-significant differences compared with fresh water (control) treatment, leading to reinstating the soil quality. Consequently, under salinity stress, the combined application motivated the faba bean vegetative growth, e.g., root length and nodulation, which reinstated the K^+^/Na^+^ ions homeostasis, leading to the lessening or equalizing of the activity level of enzymatic antioxidants (CAT, POD, and SOD) compared with the controls of both saline water and fresh water treatments, respectively. Although the irrigation with saline water significantly increased the osmolytes concentration (free amino acids and proline) in faba bean plants compared with fresh water treatment, application of PGPR or K-silicate notably reduced the osmolyte levels below the control treatment, either under stress or non-stress conditions. On the contrary, the concentrations of soluble assimilates (total soluble proteins and total soluble sugars) recorded pronounced increases under tested treatments, which enriched the plant growth, the nutrients (N, P, and K) uptake and translocation to the sink organs, which lastly improved the yield attributes (number of pods plant^−1^, number of seeds pod^−1^, 100-seed weight). It was concluded that the combined application of PGPR and K-silicate is considered a profitable strategy that is able to alleviate the harmful impact of salt stress alongside increasing plant growth and productivity.

## 1. Introduction

Faba bean (*Vicia faba* L.) is one of the major legume crops in Asia, and the Mediterranean region [1], rich in protein (25–30%) and carbohydrate content (55–60%), which leads to the arrangement of faba beans between the popular annually cultivated grain crops for human and domestic animals consumption [2]. During vegetative growth, green seeds are used in preparing fresh vegetable salad, while dry seeds are used as a cooked food; moreover, the whole plant can be used for feeding farm animals [3]. Egypt is one of the major importer countries of faba beans due to the insufficiency of local production [1]. Hence, expanding its cultivation in the newly-reclaimed lands may be a good strategy for increasing production. The biggest challenge is that most of these lands are salt-affected soils. Furthermore, faba bean, as a salt-sensitive plant, has a tolerance limit to the soil electrical conductivity (E.C.) up to 1.6 dS m^−1^. Any further increase in soil salinity significantly reduces the yield, recording a 50% yield reduction when the soil salinity level reaches 5 dS m^−1^ [4].

In arid and semi-arid regions, several interrelated factors could restrain sustainable agricultural development, such as a lack of fresh irrigation water, soil salinity, and an increase in evapotranspiration [5]. Soil salinity adversely affected soil fertility which restricted the growth and impaired crop yields [6]. Increment levels of soil salinity are attributed to the irrigation of crops with saline water [7]. It was estimated that roughly 40% of the irrigated land was influenced by salinity and 1.5 Mha falls outside of agricultural production every year, which is projected to amplify in the coming years. It is proven that crop yields are linked to soil health and water quality [8]. As the need for irrigation water increases, substitute sources are being sought. Saline irrigation water was deemed unfeasible for agricultural production [9]. Nevertheless, farmers are forced to utilize low-quality irrigation water for crop production, especially in arid and semi-arid regions [10]. Cultivation of plants under salt stress (i.e., soil salinity and saline irrigation water) deteriorated the growth and development as a consequence to change in soil physicochemical characteristics, plant morphological, physiological and biochemical traits. Salt stress triggers osmotic stress, low soil water potential, nutrient imbalance, high sodium and chloride concentrations, and oxidative stress that reduces soil quality, impairs plant growth and productivity. Therefore, it is inevitable that we must attain alternative techniques used to overcome the harmful impacts of salt stress to maximize agricultural production [11].

Globally, there is an incremental demand for a potential strategy for the application of cost-effectively promising and eco-friendly approaches in saline soil agriculture. The adverse impact of salt stress can be minimized by applying beneficial bacteria strains that are among the most promising practices to enhance soil health, plant growth, and development. These efficient bacteria are indicated as plant growth-promoting rhizobacteria (PGPR) [12]. Seed inoculation with PGPR that can colonize plant roots enhances seed germination rate [13], root growth and plant development under salt stress as a consequence of its potential in a symbiotic N_2_ fixation, and root hydraulic conductivity [14], production of plant hormones like auxins, cytokinins and gibberellins as well as production of 1-aminocyclopropane-1-carboxylate deaminase [15], maintaining a high K^+^/Na^+^ ratio, scavenging reactive oxygen species (ROS), stimulating the activity of soil enzymes [16], and enhancing soil physicochemical properties [17]. Consequently, a profitable strategy to mitigate the harmful impacts of salt stress with an increase in plant growth could be the co-inoculation of seeds with different PGPR strains [18,19].

*Rhizobia* is considered as one of the most vital PGPR for legume plants, concerning its symbiosis potential to form nodules and fix N_2_ [20]_._ Isolates from stress-affected soil are the most effective PGPR under different types of environmental conditions [21]. Faba bean growth and productivity recorded a significant increase when inoculated with *Rhizobium leguminosarum* bv. *viciae* under alkaline soil or salinity stress conditions [20,22]. *Bacillus circulans* is another PGPR that has many benefits to inoculated plants, like its ability to enrich K ion availability to salt-affected faba bean plants [23].

The application of foliar spraying on plants is an efficient approach and has become a substitute technique in agriculture used to stimulate growth and alleviate the deleterious impact against salt stress [24]. Potassium silicate (K_2_SiO_3_) is used as a plant biostimulant and a source of both potassium (K) and highly soluble silicon (Si) [25]. Silicon can be deemed as one of the most significant elements in crop production, especially in minimizing the negative impacts of salt stress and oxidative stress [26]. Despite this, the small amount of information on the effects of Si on faba bean under salt stress is still insufficient. Si enhances the plant potential under salt stress by reducing Na^+^ absorption and increasing K^+^ absorption in the leaves [27]. Hence, Si can improve root architecture, plant growth, leaf erectness, photosynthesis, and water relations. Potassium is one of the essential elements of the plant and plays a pivotal role in the formation of sugars and starch, protein synthesis, cell division, growth, seed size and quality [28]. Potassium has been shown to stimulate root length, vegetative growth, and osmoregulation and enhance physiological processes such as chlorophyll pigments, stomata movement, and water status [28]. Potassium has been proven to improve ionic balance and antioxidant enzymatic activity [29]. Hence, it can be used to minimize the harmful effect of salt stress in plants. Potassium silicate could improve yield-related traits, seed yield and quality, and nutrient (N, P, and K) uptake [24]. Some reports have shown that potassium silicate effectively affects plant development, production, and quality [30,31]. As a consequence, enhancing the activities of enzymatic antioxidants during salinity stress retains the plasma-membrane functions, e.g., controlling the permeability, which contributes to higher root activity, strengthening the root’s ability to acquire the necessary nutrients [32,33].

To date, no salt-tolerant cultivars of *Vicia faba* have been produced to cultivate in the newly-reclaimed and salt-affected soils. Consequently, a valuable strategy in this study was evaluated to mitigate the harmful impacts of salt-affected soil through the coupled application of PGPR (*Rhizobium leguminosarum* and *Bacillus circulans*) and potassium silicate (K_2_SiO_3_) on faba bean irrigated with saline water taking into account the modifications in soil properties, nodulation, soil enzyme activity, physiological traits, antioxidant enzymatic activity, biochemical attributes, seed yield, and nutrient uptake.

## 2. Results

Soil fertility and plant development were significantly minimized as a consequence of irrigation faba bean with saline water in salt-affected soil, resulting in low productivity. The dual application of PGPR and K-silicate could maximize the soil enzyme activity, nodulation, soil properties, availability of inorganic solutes, enzymatic and non-enzymatic antioxidants defense system, physiological processes, metabolism activity, yield-related traits, and nutrient uptake in faba bean compared to their individual application and untreated plants during the 2018/2019 and 2019/2020 seasons.

### 2.1. Soil Quality Indicators

#### 2.1.1. Soil Enzyme Activity

**Figure 1:**
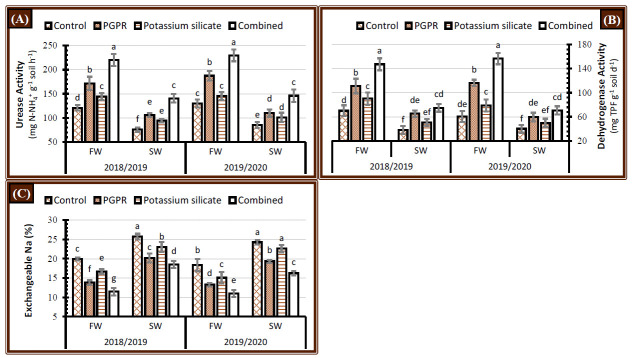
Effect of application of plant growth-promoting rhizobacteria (PGPR), potassium silicate and their combination on (**A**) urease activity and (**B**) dehydrogenase activity (**C**) exchangeable sodium percentage in faba bean under two irrigation types, i.e., fresh water (FW) and saline water (SW) in salt-affected soil during two growing seasons 2018/2019 and 2019/2020. The data are means ± SD (error bar) of four replicates. Means values that have the same lower-case letter in each column per season are not significant according to Duncan’s Multiple Range Test.

Activity of urease and dehydrogenase enzymes in the soil cultivated with faba bean plant significantly declined when plants were irrigated with saline water compared to fresh water in salt-affected soil during the 2018/2019 and 2019/2020 seasons (Figure 1). Application of PGPR or/and K-silicate as individuals or dual application significantly boosted the soil enzyme activity (urease and dehydrogenase) during both seasons, which minimized the harmful impact of irrigation with saline water (Figure 1A,B). The individual application of PGPR had a higher effect than K-silicate in increment of soil urease and dehydrogenase enzyme activity under either fresh or saline water supply. The highest activity of soil enzymes was obtained when plants were treated with the co-application of PGPR and K-silicate irrespective of the type of irrigation water during both seasons. However, the co-application of PGPR and K-silicate had more urease and dehydrogenase activity in the soil, and their levels of activities under salinity stress reached the levels of control plants irrigated with fresh water.

#### 2.1.2. Soil Exchangeable Na Percentage

Exchangeable sodium percentage (ESP) in the soil at harvest time significantly expanded when plants were irrigated with saline water in salt-affected soil during the 2018/2019 and 2019/2020 seasons (Figure 1C). Subjected faba bean plants to the sole or dual application of PGPR or/and K-silicate significantly decreased the soil ESP values during the 2018/2019 and 2019/2020 seasons (Figure 1C) which diminished the detrimental effects of salinity stress. The lowest soil ESP values were attained when the plants had treated with the synergistic application of PGPR and K-silicate, regardless of the type of irrigation water during both years (Figure 1C). The sole application of PGPR showed a higher inductive impact than K-silicate in decrement of the soil ESP under either fresh or saline water supply. Nevertheless, it was affirmed that the soil cultivated with faba bean plants which were irrigated with saline water and treated with the coupled of PGPR and K-silicate, had the lowest soil ESP value compared with control plants that were irrigated with fresh water (Figure 1C).

### 2.2. Nodules Number, Dry Weight, and Root Length

**Figure 2:**
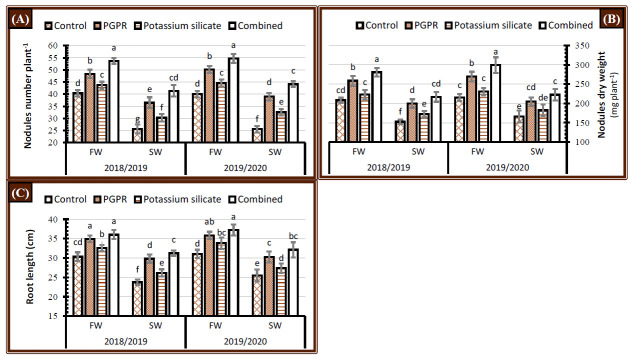
Effect of application of PGPR, potassium silicate and their combination on (**A**) nodules number, (**B**) nodules dry weight and (**C**) root length at 70 days after planting of faba bean under two irrigation types, i.e., fresh water (FW) and saline water (SW) in salt-affected soil during two growing seasons 2018/2019 and 2019/2020. The data are means ± SD of four replicates. Means values that have the same lower-case letter in each column per season are not significant according to Duncan’s Multiple Range Test.

Irrigation of faba bean plants with saline water significantly minimized nodules number, nodules dry weight, and root length than fresh water application during the 2018/2019 and 2019/2020 seasons (Figure 2). All the application forms of PGPR and K-silicate recorded higher values in nodules and root parameters over the control during the 2018/2019 and 2019/2020 seasons (Figure 2). Treated plants with the dual application of PGPR and K-silicate maximized the nodulation and root length, either for fresh watering or saline watering conditions during both seasons. The individual application of PGPR has a higher inductive impact than K-silicate application over the control treatment in boosting the nodules number, nodules dry weight, and root length under either fresh or saline water supply. Subjected faba bean plants to the dual treatment through soil application (PGPR) and foliar application (K-silicate) restored the values of nodulation and root length obtained from fresh watering control plants (Figure 2).

### 2.3. Inorganic Solutes

**Figure 3:**
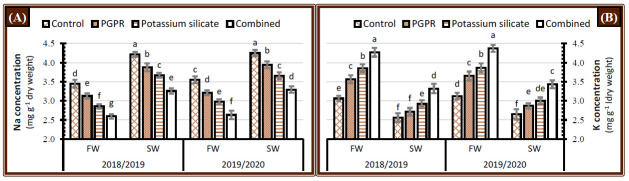
Effect of application of PGPR, potassium silicate and their combination on the concentration of (**A**) sodium ions and (**B**) potassium ions in faba bean leaves growing under two irrigation types, i.e., fresh water (FW) and saline water (SW) in salt-affected soil during two growing seasons 2018/2019 and 2019/2020. The data are means ± SD of four replicates. Means values that have the same lower-case letter in each column per season are not significant according to Duncan’s Multiple Range Test.

A maximum concentration of sodium ions (Na^+^) and a minimum concentration of potassium ions (K^+^) in the leaves of faba bean were recorded when the plants were irrigated with saline water in comparison with fresh water irrigation during the 2018/2019 and 2019/2020 seasons (Figure 3). Faba bean plants treated with the single or dual application of PGPR or/and K-silicate during the 2018/2019 and 2019/2020 seasons had a significant increase in leaf K^+^ content and a significant decrease in leaf Na^+^ content (Figure 3), which overcome the negative effects of saline water irrigation on faba bean plants grown in salt-affected soil. Regardless of the form of irrigation water used, highest K^+^ and lowest Na^+^ were obtained when plants were handled with a synergistic application of PGPR and K-silicate during both years (Figure 3). The individual application of K-silicate has a higher inductive impact than PGPR in increment of leaf K^+^ content and decrement of leaf Na^+^ content under either fresh or saline water supply. Nonetheless, the results showed that faba bean plants irrigated with saline water and treated with a combination of PGPR and K-silicate had significantly higher leaf K^+^ content and lower leaf Na^+^ content than control plants irrigated with fresh water during both seasons (Figure 3).

### 2.4. Activity of Enzymatic Antioxidants

**Figure 4:**
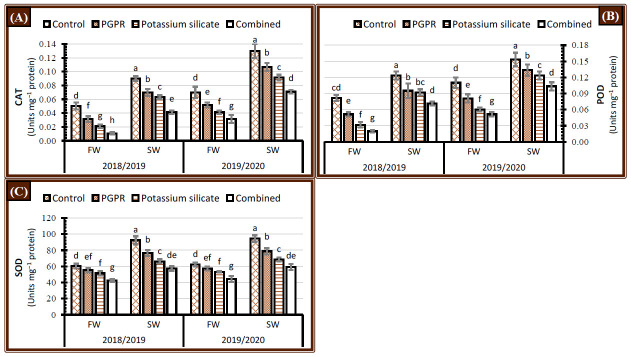
Effect of application of PGPR, potassium silicate and their combination on the activity of antioxidant enzymes (**A**) catalase (CAT), (**B**) peroxidase (POD) and (**C**) superoxide dismutase (SOD) in the leaves of faba bean plants grown under two irrigation types, i.e., fresh water (FW) and saline water (SW) in salt-affected soil during two growing seasons 2018/2019 and 2019/2020. The data are means ± SD of four replicates. Means values that have the same lower-case letter in each column per season are not significant according to Duncan’s Multiple Range Test.

Irrigated faba bean plants with saline water significantly increased the activities of enzymatic antioxidants (CAT, POD, and SOD) in comparison with fresh water irrigation during the 2018/2019 and 2019/2020 seasons (Figure 4). There was a significant decline in CAT, POD, and SOD activity when faba bean plants were treated with the individual or combined application of PGPR or/and K-silicate during both seasons. The results showed that lower enzyme activities were obtained by co-applying PGPR and K-sillicates to plants regardless of the form of water irrigation over both years (Figure 4). The individual application of K-silicate had a higher impact than PGPR over the control plants in diminution the activities of CAT, POD, and SOD enzymes under either fresh or saline water supply. Faba bean plants subjected to saline water irrigation and co-treated with PGPR and K-silicate had lower CAT, POD and SOD activity than recorded with control or individual applications under fresh water conditions (Figure 4).

### 2.5. Non-Enzymatic Antioxidants

**Figure 5:**
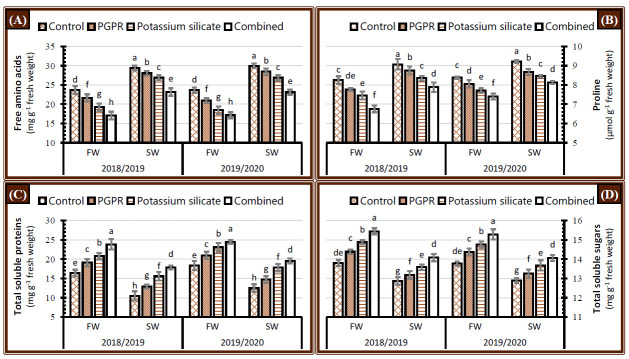
Effect of application of PGPR, potassium silicate and their combination on the concentration of biochemical constituents (**A**) free amino acid, (**B**) proline, (**C**) total soluble proteins, and (**D**) total soluble sugars in the leaves of faba bean plants grown under two irrigation types, i.e., fresh water (FW) and saline water (SW) in salt-affected soil during two growing seasons 2018/2019 and 2019/2020. The data are means ± SD of four replicates. Means values that have the same lower-case letter in each column per season are not significant according to Duncan’s Multiple Range Test.

The concentrations of total free amino acids, proline, total soluble proteins, and total soluble sugars in the leaves of faba bean plants were affected significantly after irrigation of the plants with saline water during the 2018/2019 and 2019/2020 seasons (Figure 5). Salinity stress increased the content of proline and free amino acids while total soluble proteins and total soluble sugars declined. When faba bean plants were treated with PGPR or/and K-silicate individually or in combination during the 2018/2019 and 2019/2020 seasons, there was a substantial increase in total soluble proteins and total soluble sugars whereas the opposite trend in the concentrations of proline and free amino acids were noted (Figure 5), which mitigated the negative effect of irrigation with saline water on faba bean plants grown in salt-affected soil. The results showed that when plants were treated with the synergistic application of PGPR and K-silicate regardless of irrigation water type, they had the highest total soluble proteins and total soluble sugars while having the lowest proline content and total free amino acids during both seasons (Figure 5). Individual PGPR application has a greater inductive effect than K-silicate in terms of increasing total soluble proteins and total soluble sugars, although decreasing proline and total free amino acids content under either fresh or saline water supply. Nonetheless, the results revealed in Figure 5 indicate that faba bean plants irrigated with saline water and treated with a combination of PGPR and K-silicate had significantly highest total soluble proteins and total soluble sugars while having the lowest proline and free amino acids content than plants treated with PGPR and K-silicate individually or untreated (control) plants irrigated with fresh water during both years (Figure 5).

### 2.6. Physiological Processes

**Figure 6:**
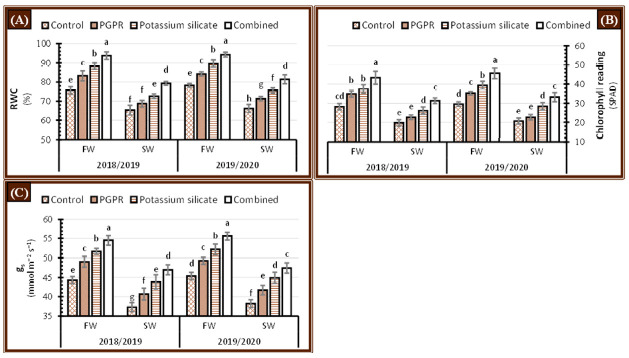
Effect of application of PGPR, potassium silicate and their combination on physiological attributes, i.e., (**A**) relative water content (RWC), (**B**) chlorophyll reading (SPAD), and (**C**) stomatal conductance (g_s_) in leaves of faba bean plants growing under two irrigation types, i.e., fresh water (FW) and saline water (SW) in salt-affected soil during two growing seasons 2018/2019 and 2019/2020. The data are means ± SD of four replicates. Means values that have the same lower-case letter in each column per season are not significant according to Duncan’s Multiple Range Test.

The indicator of chlorophyll content (SPAD reading), stomatal conductance (g_s_), and relative water content (RWC) of faba bean leaves were significantly reduced when plants were irrigated with saline water rather than fresh water in salt-affected soil during the 2018/2019 and 2019/2020 seasons (Figure 6). Significant increases in chlorophyll content, stomatal conductance, and RWC were observed when faba bean plants were treated with PGPR or/and K-silicate alone or in combination during the 2018/2019 and 2019/2020 growing seasons (Figure 6), which countered the negative effect of irrigation with saline water on faba bean plants grown in salt-affected soil. The highest chlorophyll content, stomatal conductance, and RWC were attained when faba bean plants were treated with the combination of PGPR and K-silicate regardless of the type of irrigation water during both years (Figure 6). The individual application of PGPR had a higher inductive impact over K-silicate in increment of chlorophyll content, stomatal conductance, and RWC under either fresh or saline water supply. Moreover, the single application of PGPR or K-silicate was most effective than control treatment in increasing chlorophyll content, stomatal conductance and RWC under either fresh or saline water supply. Nevertheless, the results shown in Figure 6 demonstrate that faba bean plants subjected to saline water irrigation and treated with a combination of PGPR and K-silicate had significantly higher chlorophyll content, stomatal conductance, and RWC than the individual applications and control plants that were irrigated with fresh water during both years.

### 2.7. Yield and Yield-Related Traits

Number of pods plant^−1^, number of seeds pod^−1^, 100-seed weight (g), and seed yield (t ha^−1^) in faba bean significantly minimized when plants were irrigated with saline water compared to fresh water in salt-affected soil during the 2018/2019 and 2019/2020 seasons (Table 1). There was a significant increment in the number of pods plant^−1^, number of seeds pod^−1^, 100-seed weight (g), and seed yield (t ha^−1^) when faba bean plants were treated with the individual or dual application of PGPR or/and K-silicate during 2018/2019 and 2019/2020 seasons. The combined application of PGPR and K-silicate maximized the number of pods plant^−1^, number of seeds pod^−1^, 100-seed weight (g), and seed yield (t ha^−1^) regardless of the type of irrigation water during both years (Table 1). Individual applications of PGPR had a greater inductive effect than K-silicate in terms of increasing the number of pods plant^−1^, the number of seeds pod^−1^, the 100-seed weight (g), and the seed yield (t ha^−1^) under either fresh or saline water supply. Additionally, the combination of PGPR and K-silicate reinstated the values of number of pods plant^−1^, number of seeds pod^−1^, 100-seed weight (g) and seed yield (t ha^−1^) over control treatment irrigated with fresh water in both years (Table 1).

### 2.8. Grain N, P, and K Uptake

Faba bean grain N, P, and K uptake was significantly minimized when irrigated with saline water during the 2018/2019 and 2019/2020 seasons (Table 2). When faba bean plants were treated with the single or dual application of PGPR or/and K-silicate during the 2018/2019 and 2019/2020 seasons (Table 2), there was a substantial increase in grain N, P, and K uptake, which mitigated the negative effect of irrigation with saline water on faba bean plants grown in salt-affected soil. Higher grain N, P, and K uptake was observed in salt-affected soil when plants were treated with a synergistic application of PGPR and K-silicate regardless of irrigation water type throughout both years (Table 2). The individual application of PGPR showed a higher inductive impact than K-silicate in increment of grain N, P, and K uptake under either fresh or saline water supply. The dual application of PGPR and K-silicate had significantly maximized grain N, P, and K uptake over the individual applications and control plants that were irrigated with fresh water in both years in salt-affected soil (Table 2).

## 3. Discussion

Irrigation of field crops with low-quality water in salt-affected soil increases growth inhibitors and decreases growth promoters, resulting in reduced soil quality, ionic imbalance, water disturbance, accumulation of ROS, deteriorated physiological processes, and biochemical constituents, and accordingly, cell death and low productivity. In this study, a cost-effective and eco-friendly fruitful strategy was used to reduce the adverse effect of saline water used in irrigating faba bean plants grown in salt-affected soil using the PGPR, K-silicate, or their combination to improve soil quality and plant growth and productivity.

### 3.1. Soil Quality Indicators

The activity of soil enzymes is related to soil organic matter and its microbial content. Any unfavorable change on the soil structure and properties, like irrigation of the soil with saline water, leading to a marked reduction in the activities of soil dehydrogenase and urease (Figure 1A,B). This decline could refer to the osmotic effect of salinity on the soil nutritional imbalance, which is subsequently reflected on the metabolism and enzyme synthesis of soil microbes [34]. Exogenous application of K-silicate has a positive role in enhancing plant growth, root length, and architecture [28,35], which mitigates the deleterious effect of salinity in the faba bean rhizosphere leading to increasing microbial biomass, which indirectly reflecting on prompting the synthesis of soil enzymes. Moreover, K-silicate could stimulate the availability of soil nutrients, such as N, which has a direct positive relationship with the activities of soil enzymes [36,37]. The direct application of PGPR to the soil or in the inoculated seeds greatly increases microbial biomass and community diversity, which influenced the soil enzymes [38]. In this regard, the dual application of K-silicate and PGPR maximized the soil health status.

Irrigation with saline water negatively impacts the soil Na^+^ content, which directly increases the soil Exchangeable sodium percentage (ESP) (Figure 1C). High Na^+^ competes with K^+^ due to antagonistic impact between Na^+^ and other ions such as Ca^2+^, Mg^2+,^ and K^+^, resulting in the displacement of Ca^2+^ in the root cell membrane. The decline in Ca^2+^ and Mg^2+^ absorption under salt stress could be attributed to the exploitive impact of Na^+^ on these cations and the declined transport of Ca^2+^ and Mg^2+^ ions [39]. Subsequently, high K^+^ content in the soil and retaining a low Na^+^ content are imperative for plant growth [39]. Soil organic amendments positively enhanced the salt-affected soil and decreasing ESP values [40]. Furthermore, the application of PGPR excreted polysaccharides from soil microbes which increased the availability of nutrients [41], alongside endogenous phytohormones such as IAA that lessened the ESP significantly, resulted in improvement of CEC that holds essential nutrients and soil physicochemical properties irrigated with saline water [42]. Thus, the combined effect of K-silicate and PGPR on salt-affected soil, alongside their positive impact on soil enzyme activities, maximized the reduction of ESP, leading to the effective soil quality characteristics (Figure 1).

### 3.2. Nodulation and Root Length

Reduction in faba bean growth rate under salinity stress in the form of root length is extensively reported, which refers to the sensitivity of faba bean to abiotic stresses (Figure 2C). Symbiosis efficiency between rhizobacteria and legumes roots is much related to soil conditions. Adverse effects of salinity on root length, parallelly recorded for nodules number and nodules dry weight (Figure 2A,B). which could be related mainly to the effect of osmotic stress on the development of both *Rhizobium* and root cells, which reflected on nodulation. Application of Si (K-silicate) strengths the development of root cells [43,44], which reflecting on enhancing the nodules number [45]. This positive impact can be ascribed to the enhancing role of potassium and silicate on cell transport systems that transport nutrients and water in addition to augmenting water permeability and water retaining [46]. It was found that the application of silicon declines Na^+^ absorption, prohibited Na^+^ deposition in the roots under salt stress, and increased K^+^ absorption. Foliar application of K-silicate stimulated root growth and nodulation, which promoted nutrients and water uptake [47]. This can be attributed to the increase in root elongation, lateral roots, and consistent with the signaling pathway for plant hormones which stimulated the growth [48,49]. Inoculation using *Rhizobium leguminosarum* biovar *viciae* (TAL–1148) and *Bacillus circulans* NCAIM B.02324 has excellent potential for nitrogen cycling, which in turn increase soil urease and dehydrogenase activities (Figure 1). The maximum soil enzyme activity can be associated with heavier nodules and deeper root length due to the enhancing of root and plant growth through soil nutrient enrichment, which improves the root exudates in the rhizosphere and consequently enlarging the microbial community [30,43]. The inoculation of PGPR strains positively affected modifications in root morphogenesis and augmented lateral root length and the density of root hairs, which are closely associated with the production of phytohormones, including auxins, cytokinins, and gibberellins [44]. Higher numbers and dry weight of nodules in our study could be ascribed to the stimulating impacts of PGPR application on root hair formation and root length that increase the efficiency of nutrient and water absorption under salt-affected soil [50].

### 3.3. Inorganic Solutes

Irrigation of faba bean plants with saline water raised the concentrations of salts that exist in the root region, which impaired plant cells due to the accumulation of excess amounts of Na^+^ ions in leaf cells (Figure 3). Moreover, increasing soil osmotic pressure and toxic ions are consequences of Na^+^-derived salinity that lessens water absorption and soil enzyme activity [9]. Consequently, soil solution including high contents of Na^+^ and Cl^−^ impedes K^+^, Ca^2+^ absorption resulting in the nutritional disorder [10]. Application of PGPR strains promoted the equilibrium of ions in the rhizosphere region that reduced Na^+^ absorption and increased K^+^ absorption from the soil solution through the roots to leaves in faba bean plants [11]. This can be ascribed to the excretion of IAA and bacterial exopolysaccharide, which can bind to Na^+^ and reduce its absorption [13]. Foliar application of K-silicate sustained normal metabolic responses, managing an increase in K^+^ uptake and a decrease in Na^+^ influx in the leaf tissues, findings that are in accordance with those of Ibrahim et al. [50]. Higher K^+^ uptake and lower Na^+^ in leaf tissues could be one of the feasible mechanisms of salt tolerance by K-silicate addition in faba bean plants. Combination treatment of K-silicate and PGPR has a chelating impact on nutrients that cause the uptake and transportation of nutrients faster, up to its impact on cell membrane permeability [31]. So, the coupled impact of applied PGPR and K-silicate has the potential to maximize K^+^ uptake and minimize Na^+^ content compared with that of sole application in faba bean plants irrigated with saline water.

### 3.4. Activity of Enzymatic Antioxidants

Under abiotic stresses, plants extend the activity of antioxidant enzymes to their full potentiality depends on plant stress-sensitivity status as a fast defense line to cope with the excess amounts of ROS [51,52]. Results in Figure 4 are in harmony with this conclusion, where the activity of antioxidant enzymes (CAT, POD, and SOD) boosted over the control when irrigated with saline water. Reducing the leaves Na^+^ ions content under tested treatments (K-silicate, PGPR, and combination) in the irrigated plants with saline water (Figure 3), may be the main reason behind the observed decline in the activity of CAT, POD, and SOD in the leaves (Figure 4). On the contrary, Qados [53] reported that silicon application increased the activity of antioxidant enzymes under salinity stress, which could be attributed to the differences in the physiological age of leaves samples. Additionally, the application of PGPR positively enhanced the activity of antioxidant enzymes in the roots via removing H_2_O_2_ from salt-stressed roots and impeding the production of OH^−^ radicals, which declined the oxidative stress [54]. In this regard, the shoot system may be less exposed to the deleterious effects of salinity stressed plants inoculated with PGPR, leading to declining enzyme activity compared with non-treated plants (Figure 4). From this point of view, the lowest enzyme activity values were for the coupled PGPR and K-silicate treatment, which could be indirectly referred to as reinstating the normal metabolic status for the treated plants compared with sole or control treatments, especially under salinity stress.

### 3.5. Non-Enzymatic Antioxidants

Micro-osmo-protectants (free amino acids, proline, and monosaccharides) repeatedly increase in many plants as a mitigation response to osmotic stresses directed from drought or salinity [55,56,57]. In this regard, significant increases in the concentration of total free amino acids and proline were recorded in faba bean plants irrigated with saline water (Figure 5A,B). Meanwhile, the concentration of macro-osmo-protectant (soluble polysaccharides) and total soluble proteins were reduced under the same conditions (Figure 5C,D), which could be attributed to catabolic reactions related to stress-tolerance pathways, leading finally to a degradation in the macro-molecules (proteins and sugars) to its assimilates, or even reduce the anabolic reactions and conversion of the assimilates (free amino acids, proline, and monosaccharides) to the equivalent macro-molecules [56]. Additionally, irrigation of faba bean plants with saline water caused modifications in biochemical constituents of salinity stressed faba bean plant alongside with the lessening in physiological processes which restrained photosynthetic activity, a decline of carbon dioxide in intercellular spaces of stomata, lowering the level of 3-phosphoglycerate and reducing the starch synthesis [58,59]. PGPR application promoted the synthesis of total soluble proteins and total soluble sugars, meanwhile decreased the levels of proline and free amino acids in faba bean plants irrigated with saline water, which may be directed from the surplus hormones synthesized by PGPR, leading to an increase in the levels of endogenous phytohormones like IAA and cytokinins, which stimulate the developmental processes related to anabolic pathways, like carbohydrates and proteins synthesis [30,60]. The foliar application of K-silicate promoted the concentrations of proline, amino acid, soluble protein, and soluble sugars, in addition to the biosynthesis of endogenous hormone that promoted cell division and cell enlargement of faba bean plants [61], an increase in nutrient uptake [62], IAA and cytokinins production [63]. The magnitude increase in the biochemical constituents was more pronounced with the coupled application of PGPR and K-silicate.

### 3.6. Physiological Processes

Decreases in the leaf relative water content are considered the first known symptom in the plants subjected to osmotic stresses (Figure 6A), which is possibly due to the surplus amounts of ions in saline water negatively reducing the osmotic potential of soil solution, which alters the mass flow water movement system from soil solution to the root xylem vessels, which are controlled mainly by differences in water potential values between soil solution and cell sap. As a consequence, water absorption by roots from the soil will reduce, which directly decreases the leaf cells’ turgor and RWC. Moreover, irrigation with saline water decomposes the chlorophyll pigment (Figure 6B), which could be related to the oxidation effect by ROS formed under salinity stress, causing damage to chloroplast ultrastructure [64]. Stomatal conductance significantly reduced under saline water treatment (Figure 6C), which linked to stomatal closure to maintain tissue water content under osmotic stress to control transpiration rate. [65]. Application of PGPR significantly mitigated the harmful impact of NaCl in the soil solution which increased K uptake to leaf tissues, maintained essential nutrients flow from roots to plant leaves and water holding capacity resulted in improvement of the assimilate translocation and biosynthesis as well as osmoregulation and protection of leaf tissues from salinity-induced oxidative stress [66]. Foliar-applied of K-silicate has a more pronounced ameliorative impact on faba bean plants irrigated with saline water [58]. Foliar application of K-silicate is considered the source of potassium and silicon elements that plays a vital role in sustaining and improving a plant’s photosynthetic apparatus as a result of enhancing leaves number and area [67]. Foliar-applied of K-silicate regulates the stomatal conductance, promoting ATPase, DNA and RNA synthesis as well as maintains the ionic balance in faba bean leaves irrigated with saline water [68].

### 3.7. Yield Traits and Nutrients Uptake

Flowering, reproductive growth, and interconnected yield attributes are the most sensitive growth stage susceptible to detrimental changes in environmental conditions. In addition, fruit and seed formation as the end products in the plant life cycle are dependent mainly on vigorous vegetative growth. Salinity stress imbalanced the soil nutrients status as a result of overflowing Na^+^ ions, which lowered the uptake of other minerals, particularly of K^+^ (Figure 1). Subjected faba bean plants at seedling and vegetative growth stages to osmotic stress directed from irrigation with saline water, decreased root growth and nodulation (Figure 2), K concentration (Figure 3), which led to lower leaf water content and its involved physiological functions; photosynthetic pigments and stomatal conductance (Figure 6), which decreased the assimilation rate and its related synthesized molecules, total soluble proteins and sugars (Figure 5), and the uptake of N, P, and K (Table 2), reflecting finally on the efficiency of biomass production and declined the yield attributes (Table 1); number of pods plant^−1^, number of seeds pod^−1^, 100-seed weight (g) and seed yield (t ha^−1^). This could be a consequence of the reduction in transpiration rate, ion imbalance, osmotic stress and reduction in the uptake of water and nutrients [69]. PGPR application had a beneficial impact on the yield-related traits and productivity of faba bean. The promising impacts of PGPR on plant yield might be ascribed to their efficiency in producing phytohormones [41] and polysaccharides alongside solubilizing phosphate and a symbiotic N_2_ fixation that increased the physiological processes and improved the biochemical constituents [42] resulting in increased yield-related traits and productivity in faba bean plants irrigated with saline water. However, foliar-applied K-silicate could alleviate salt stress through augment leaf area [31], cell enlargement and enhance the capacity of photosynthetic machinery, which is closely related to stabilizing and maintaining physiological properties [24]. These data were in line with previous investigations, which confirmed that K-silicate could stimulate water holding capacity and uptake of inorganic nutrients such as P, K, Ca, and Mg in the plant parts and hinder Na influx which enhances the intercellular CO_2_ concentration and leaf water content [58]. It was confirmed that K-silicate improved the transport of assimilates and associated N-metabolism traits from leaves to seeds to support seed growth and leveling up the content of proteins and carbohydrates in the seeds [61,63,70]. The extent increase in yield traits was more pronounced with the paired application of PGPR and K-silicate.

## 4. Materials and Methods

### 4.1. Plant Material and Experimental Detail

The purpose of the study was to investigate the effect of seed inoculation of PGPR (*Rhizobium* and *Bacillus*) and foliar application of potassium silicate (K_2_SiO_3_) on soil enzyme activity, soil properties, growth traits, inorganic solutes, physiological processes, biochemical constituents, antioxidant enzymes and yield as well as the nutrient content of faba bean (*Vicia faba* L., cv. 716) in salt-affected soil. A two-seasonal experiment was performed at the Sakha Agricultural Research Station (SARS) Farm, Kafr El-Sheikh, Egypt during two consecutive winter growing seasons of 2018/2019 and 2019/2020. Experimental design was a split-plot design arranged in randomized complete blocks with four replicates. Faba bean plants were subjected to eight treatments including, two types of irrigation water (fresh water and saline water) were allocated in the main plots, while soil and foliar treatments (control, PGPR, K-silicate and combined PGPR + K-silicate) were allocated in the sub-plots. The experiment began from November 2018 and continued until April 2019 and repeated in the successive season. Each plot (3 × 4 m) consisted of five ridges 4 m in length and 60 cm apart; the seeds were planted at a rate of 2–3 seeds per hole with 20 cm spacing in between and the space between replications was 1 m. Seeds of faba bean were provided by the Leguminous Research Department, Sakha Agricultural Research Station, Kafr El-Sheikh, Egypt. Seeds were placed into one side of ridges at 85 kg ha^−1^ as recommended. The thinning process for plants was carried out directly prior to the first irrigation to ensure one plant per hill. All treatments received the recommended dose of nitrogen, phosphate and potassium fertilization at the rate of 50 kg N ha^−1^, 80 kg P_2_O_5_ ha^−1^ and 114 kg K_2_O ha^−1^, respectively before planting.

The chemical characteristics of irrigation water are presented in Table 3, meanwhile, the soil physico-chemical traits are described in Table 4. After 70 days of begin from the trial, ten plants were randomly harvested from each treatment for further analysis.

### 4.2. PGPR and Potassium Silicate

The association of two rhizobacterial strains of *Rhizobium leguminosarum* bv. *viciae* (TAL–1148) and *Bacillus circulans* NCAIM B.02324 were prepared. These strains were acquired from Agricultural Microbiology Department, Soils, Water and Environment Research Institute (SWERI), Agricultural Research Centre (ARC), Egypt. Pure cultures of *Rhizobium* and *Bacillus* were routinely maintained on Yeast Extract Mannitol Broth (YEMB) medium and Nutrient Broth (NB) medium, respectively [71]. Before planting, seeds were inoculated by a mixture (1:1) of the two PGPR strains (prepared by mixing 15 mL of 1 × 10^8^ CFU mL^−1^ from each culture to 13 gm of the sterilized carrier) at a rate of 1000 g ha^−1^. Faba bean plants were sprayed with 300 mg L^−1^ K-silicate (K_2_SiO_3_) at 30, 45 and 60 days from planting.

### 4.3. Measurements

#### 4.3.1. Soil Enzyme Activity

Urease and dehydrogenase enzyme activity in the soil were estimated according to the method of Kandeler [72] and Mersi [73], respectively. Soil samples were collected at 70 days after planting 0–30 cm soil depth. Assessment of urease activity was calculated according to the quantitative estimation of ammonia concentration of the soil extract was measured spectrophotometrically at 660 nm [72]. Assessment of dehydrogenase activity was calculated after the incubation of soil with a colorless, water-soluble substrate, INT (2-(p-iodophenyl)-3-(p-nitrophenyl)-5-phenyl tetrazolium chloride solution), for 2 h at 40 °C, followed by extraction of the reduced INTF (iodonitro-tetrazolium formazan) from the soil with ethanol and was assessed at a wavelength of 464 nm using UV-160A spectrophotometer (Shimadzu, Japan) [73].

#### 4.3.2. Soil Exchangeable Na Percentage Determination

At harvesting time of faba bean, soil samples were selected from each treatment type 0–30 cm soil depth using an auger. Soil samples were air-dried and passed through a 2-mm mesh to assess soil Na^+^, Ca^2+^ and Mg^2+^ contents in paste extract using Atomic Absorption Spectrophotometer (AAS 3300, Perkin Elmer Ltd., UK) to compute soil sodium adsorption ratio (SAR). Then, ESP was assessed according to the equation proposed by Seilsepour et al. [74]:

ESP = 1.95 + 1.03 × SAR (R^2^ = 0.92)

#### 4.3.3. Nodulation and Roots Assessment

Nodules number, dry weight and root length were measured at 70 days after sowing. Ten plant samples were collected randomly from each treatment type and uprooted by a shovel in order to attain whole roots and nodules in 30 cm soil depth. The remaining soil on the roots was gently eliminated and the roots with intact nodules were rinsed carefully by tap water on a metal mesh sieve. The nodules on the roots were detached, pooled and oven-dried at 65°C until stabilization of its dry weight, then nodules number and dry weight were determined by an electronic balance. Root length was measured using a ruler.

#### 4.3.4. Inorganic Solutes Assessment

The content of K^+^ and Na^+^ ions in the plant leaves was estimated at 70 days after planting by Atomic Absorption Spectrophotometer (AAS 3300, Perkin Elmer Ltd., Buckinghamshire, UK).

#### 4.3.5. Assessment the Activities of Antioxidant Enzymes

At 70 days after planting, activities of catalase (CAT), peroxidase (POD) and superoxide dismutase (SOD) were determined at the second fully-expanded leaf from the plant top according to methods described by Aebi [75], Vetter et al. [76], and Beauchamp and Fridovich [77], respectively. The leaves samples (0.6 g each) were mashed in 5 mL of 50 mM cold K-phosphate buffer. The homogenates were centrifuged for 20 min at 10,000× *g* at 4 °C. Supernatant was used to determine the antioxidant enzyme activity as (Units mg^−1^ protein).

#### 4.3.6. Osmo-Protectants and Soluble Protein Determination

Concentrations of proline in the leaves were determined using the acid-ninhydrin reagent and read at 520 nm as described by Bates et al. [78]. Total soluble sugars (TSS) were extracted and estimated according to the method of Dubois et al. [79]. Briefly, 0.1 mL of the ethanolic extract was added to 3 mL of anthrone reagent and incubated in a hot water bath for 10 min. The absorbance of the mixture was recorded at 625 nm and total soluble sugars were calculated as mg g^−1^ FW. Leaf soluble protein content was extracted and assessed using the Folin phenol reagent according to the method described by Lowry et al. [80]. Total free amino acids were extracted from leaves samples using 80% hot ethanol using the method of Irigoyen et al. [81]. Concentration of total free amino acids was determined using ninhydrin reagent according to the method described by Swamy [82]. At 570 nm, the formed purple color was reported.

#### 4.3.7. Physiological Processes Assessment

All physiological parameters were estimated between 9:00 and 11:00 AM, using the central leaflet of the second fully expanded leaf from the plant top at 70 days after planting for each treatment type. Chlorophyll readings were measured from the fully expanded leaf using the soil plant analysis development (SPAD) meter (Model: SPAD-502, Minolta Camera, Osaka, Sensing Ltd., Japan) and their average to have a single value for a plant to measure leaf greenness which was determined by Ling et al. [83]. Observations concerning the stomatal conductance (g_s_, in mmol m^−2^ s^−1^) were estimated using dynamic diffusion porometer (Delta-T AP4, Delta-T Devices Ltd., Cambridge, UK), from the topmost fully expanded leaf on sunny days. Leaf relative water content (RWC) was assessed using the equation proposed by Weatherley [84], RWC (%) = [(Fresh weight–Dry weight)/(Saturated weight–Dry weight)] × 100. The selected fresh leaves were weighed to obtain fresh weight, followed by immersion of the leaves segments in distilled water for 4 h, to measure the saturated weight, then leaves oven-dried to record the dry weight.

#### 4.3.8. Yield Attributes Assessment

Yield and its related traits were determined at the end of the physiological maturation stage. Ten random faba bean plant samples were harvested from each treatment type and sun-dried, and then seed threshing was operated manually from their pods. Seeds from each plant were counted to estimate the mean values of number of pods plant^−1^ and number of seeds pod^−1^, which were pursued by weighing to estimate 100-seed weight (g) and seed yield (t ha^−1^) at 12% moisture content.

#### 4.3.9. Assessment of Nitrogen, Phosphorus and Potassium Uptake

At harvesting time of faba bean, Air-dried seeds samples from each treatment type were oven-dried for 48 h on 70 °C and ground, then digested with HNO_3_:HClO_4_ solution (2:1) to estimate N, P, and K uptakes. The concentration of N was measured by the modified macro-Kjeldahl method, while P and K concentrations were measured using Atomic Absorption Spectrophotometer as per the procedures proposed by [85]. The calculations of N, P, and K uptakes were performed according to the following equations:

$$\mathrm{Seeds}\;N\;\mathrm{uptake}\;\left(\mathrm{kg}\;{\mathrm{ha}}^{-1}\right)=\frac{\mathrm{Seed}\;N\;\mathrm{conc}.\left(g\;{\mathrm{kg}}^{-1}\right)\times \mathrm{Seed}\;\mathrm{dry}\;\mathrm{yield}\;\left(\mathrm{kg}\;{\mathrm{ha}}^{-1}\right)}{1000}$$

$$\mathrm{Seeds}\;P\;\mathrm{uptake}\;\left(\mathrm{kg}\;{\mathrm{ha}}^{-1}\right)=\frac{\mathrm{Seed}\;P\;\mathrm{conc}.\left(g\;{\mathrm{kg}}^{-1}\right)\times \mathrm{Seed}\;\mathrm{dry}\;\mathrm{yield}\;\left(\mathrm{kg}\;{\mathrm{ha}}^{-1}\right)}{1000}$$

$$\mathrm{Seeds}\;K\;\mathrm{uptake}\;\left(\mathrm{kg}\;{\mathrm{ha}}^{-1}\right)=\frac{\mathrm{Seed}\;K\;\mathrm{conc}.\left(g\;{\mathrm{kg}}^{-1}\right)\times \mathrm{Seed}\;\mathrm{dry}\;\mathrm{yield}\;\left(\mathrm{kg}\;{\mathrm{ha}}^{-1}\right)}{1000}$$

### 4.4. Data Analysis

Data of the two seasons were implemented in SPSS 13.0 software package (SPSS Inc., Chicago, IL, USA), and separately analyzed for each season. The data were exposed to analysis of variance (ANOVA) to test the significant differences between exogenous soil and foliar treatments, irrigation water type and their interactions at *P* < 0.05, followed by Duncan’s multiple range test [86]. Data are presented in the figures as bars (±error bar) which indicate to means (±SD).

## 5. Conclusions

Referring to the significant decline in the soil health and biomass of faba bean plants, irrigated with saline water in salt-affected soil, it is concluded that faba bean cv. 716 is a salt-sensitive plant that needs new strategies to deal with such types of cultivation conditions. Individual application of PGPR or K-silicate boosted the faba bean physiological traits which mitigated the negative impact of salinity on yield-related traits. Synergistic application of PGPR (soil application) and K-silicate (foliar application) expanded the positive results to the uppermost levels, especially for soil health, balance of mineral nutrition, antioxidants defense system, integrity of photosynthetic machinery and related synthesized assimilates which reinstated the plant growth and yield traits to its level under fresh water irrigation. It could be suggested that the production of the faba bean plant cultivated in salt-affected soil using the combined application of PGPR and K-silicate is a fruitful strategy with immense benefits such as cost-effectivity and eco-friendliness, particularly in arid and semi-arid regions.

## Figures and Tables

**Table 1:** Yield and yield-related traits of faba bean plants irrigated with fresh and saline water in salt-affected soil in presence of PGPR and potassium silicate during two growing seasons.

Treatments			Pods No. Plant^−1^	Seeds No. Pod^−1^	100-Seed Weight (g)	Seed Yield (t ha^−1^)
2018/2019	FW ^‡^	Control	15.5 ± 0.9 e	4.5 ± 0.36 cd	54.3 ± 2.1 e	2.2 ± 0.10 d
		PGPR	19.7 ± 0.8 c	5.7 ± 0.42 b	65.5 ± 2.4 c	2.8 ± 0.04 bc
		K silicate ^†^	22.8 ± 0.8 b	6.1 ± 0.56 b	76.7 ± 2.7 b	3.1 ± 0.17 b
		PGPR + K silicate	28.3 ± 1.2 a	6.7 ± 0.15 a	83.7 ± 2.6 a	3.6 ± 0.21 a
	SW ^§^	Control	10.7 ± 1.2 g	3.2 ± 0.20 e	32.3 ± 1.3 h	1.5 ± 0.18 f
		PGPR	12.9 ± 1.7 f	4.1 ± 0.17 d	40.8 ± 1.4 g	1.7 ± 0.22 ef
		K silicate ^†^	14.4 ± 1.2 ef	4.3 ± 0.14 d	47.7 ± 1.4 f	2.1 ± 0.16 de
		PGPR + K silicate	17.6 ± 1.1 d	5.0 ± 0.39 c	57.3 ± 1.8 d	2.7 ± 0.25 c
2019/2020	FW ^‡^	Control	17.7 ± 1.3 de	4.9 ± 0.20 de	55.8 ± 2.7 d	2.4 ± 0.12 d
		PGPR	20.2 ± 1.6 c	5.7 ± 0.21 c	66.4 ± 5.9 c	3.0 ± 0.15 bc
		K silicate ^†^	24.9 ± 2.0 b	6.2 ± 0.19 b	79.5 ± 1.4 b	3.2 ± 0.23 b
		PGPR + K silicate	29.7 ± 1.6 a	6.8 ± 0.27 a	85.5 ± 2.9 a	3.9 ± 0.17 a
	SW ^§^	Control	11.4 ± 1.4 f	3.3 ± 0.3 g	33.6 ± 2.7 g	1.6 ± 0.14 f
		PGPR	13.5 ± 1.8 f	4.4 ± 0.19 f	44.2 ± 1.6 f	2.0 ± 0.14 e
		K silicate ^†^	15.7 ± 1.1 e	4.6 ± 0.22 ef	49.6 ± 1.8 e	2.3 ± 0.13 de
		PGPR + K silicate	18.8 ± 1.0 cd	5.2 ± 0.20 d	59.3 ± 2.0 d	2.7 ± 0.16 c
Water treatments			***	**	**	**
Soil and foliar treatments			***	***	***	***
Interaction			*	ns	**	ns

Means of the same growing season designated with different lower-case letters indicate significant differences among treatments according to the Duncan’s Multiple Range test (*P* < 0.05). Values are means ± standard deviation (SD) from four replicates (Means ± SD). ***, **, *, and ns denote significance at *P* < 0.001, *P* < 0.01, *P* < 0.05, and non-significant, respectively. ^†^ Potassium silicate.; ^‡^ Fresh water.; ^§^ Saline water.

**Table 2:** Grain N, P and K uptake of faba bean plants irrigated with fresh and saline water in salt-affected soil in the presence of PGPR and potassium silicate during two growing seasons.

Treatments		N Uptake (kg ha^−1^)		P Uptake (kg ha^−1^)		K Uptake (kg ha^−1^)	
		2018/2019	2019/2020	2018/2019	2019/2020	2018/2019	2019/2020
FW ^‡^	Control	108.2 ± 2.3 e	112.4 ± 2.3 e	16.4± 0.5 e	16.9 ± 0.4 e	17.7 ± 0.6 e	19.8 ± 0.4 f
	PGPR	119.8 ± 2.5 c	123.1 ± 2.5 c	17.8 ± 0.7 c	18.0 ± 0.6 c	24.1 ± 0.5 c	26.1 ± 0.6 c
	K silicate ^†^	126.6 ± 2.9 b	129.7 ± 2.8 b	18.4 ± 0.8 b	18.9 ± 0.7 b	29.2 ± 0.7 b	30.3 ± 0.6 d
	PGPR + K silicate	138.1 ± 3.2 a	142.4 ± 2.9 a	20.2 ± 0.9 a	21.3 ± 0.9 a	36.3 ± 0.8 a	39.6 ± 0.7 a
SW ^§^	Control	86.4 ± 2.4 h	91.4 ± 2.1 h	14.1 ± 0.3 h	15.6 ± 0.5 h	6.6 ± 0.5 h	8.6 ± 0.5 h
	PGPR	95.8 ± 2.6 g	100.3 ± 2.3 g	14.9 ± 0.5 g	16.2 ± 0.6 g	9.6 ± 0.7 g	11.8 ± 0.6 g
	K silicate ^†^	104.0 ± 2.8 f	108.4 ± 2.7 f	15.6 ± 0.7 f	16.9 ± 0.7 f	13.7 ± 0.8 f	16.9 ± 0.8 e
	PGPR + K silicate	112.4 ± 2.9 d	119.4 ±3.2 d	16.9 ± 0.9 d	17.5 ± 0.8 d	19.8 ± 0.9 d	22.0 ± 0.9 d
Water treatments		***		**		***	
Soil and foliar treatments		***		***		***	
Interaction		ns		ns		*	

Means of the same growing season designated with different lower-case letters indicate significant differences among treatments according to Duncan’s Multiple Range test (*P* < 0.05). Values are means ± standard deviation (SD) from four replicates (Means ± SD). ***, **, *, and ns denote significance at *P* < 0.001, *P* < 0.01, *P* < 0.05, and non-significant, respectively. ^†^ Potassium silicate.; ^‡^ Fresh water.; ^§^ Saline water.

**Table 3:** Characteristics of irrigation water used in the two winter seasons 2018/2019 and 2019/2020.

Character	Fresh Water		Saline Water	
	2018/2019	2019/2020	2018/2019	2019/2020
pH	7.28	7.34	8.36	8.39
EC (dS m^−1^)	0.59	0.56	3.43	3.57
SAR	1.46	1.42	7.69	7.78
Na^+^ (meq L^−1^)	1.88	1.97	16.44	16.31
Cl^−^ (meq L^−1^)	3.40	3.28	11.52	11.70
SO_4_^2−^ (meq L^−1^)	0.12	0.13	7.83	8.13
NH_4_^+^ (meq L^−1^)	1.74	1.80	2.02	2.13

**Table 4:** Soil physicochemical traits in the two winter seasons 2018/2019 and 2019/2020.

Soil Traits	2018/2019	2019/2020
clay %	49.40	54.00
Sand %	15.53	13.00
Silt %	35.07	33.00
Soil texture	Clayey	Clayey
pH (1:2.5 water suspension)	8.24	8.02
EC (dS m^−1^)	5.52	5.24
Organic matter	1.19	1.29
Available P mg kg^−1^	9.44	8.54
Available N mg kg^−1^	9.40	8.60
Available K mg kg^−1^	309	294
Cations (meq L^−1^)		
Ca^2+^	5.18	4.91
Mg^2+^	1.34	1.22
Na^+^	16.00	19.00
K^+^	0.41	0.39
Anions (meq L ^−1^)		
HCO_3_^−^	4.13	4.10
Cl^−^	17.10	20.00
SO_4_^2−^	1.70	1.42
CO_3_^−^	0.00	0.00
